# Change of glucometabolic activity per PSMA expression predicts survival in mCRPC patients non-responding to PSMA radioligand therapy: introducing a novel dual imaging biomarker

**DOI:** 10.3389/fmed.2023.1339160

**Published:** 2024-01-17

**Authors:** Caroline Burgard, Jakob Engler, Arne Blickle, Mark Bartholomä, Stephan Maus, Andrea Schaefer-Schuler, Fadi Khreish, Samer Ezziddin, Florian Rosar

**Affiliations:** Department of Nuclear Medicine, Saarland University—Medical Center, Homburg, Germany

**Keywords:** PSMA—prostate-specific membrane antigen, PET/CT, radioligand therapy, prostate cancer, dual imaging

## Abstract

**Purpose:**

The value of [^18^F]fluorodeoxyglucose ([^18^F]FDG) PET/CT in monitoring prostate-specific membrane antigen (PSMA) targeted radioligand therapy (RLT) is still unclear. The aim of this study was to identify appropriate prognostic dynamic parameters derived from baseline and follow-up [^18^F]FDG and dual [^18^F]FDG/[^68^Ga]Ga-PSMA-11 PET/CT for monitoring early non-responding mCRPC patients undergoing PSMA-RLT.

**Methods:**

Twenty-three mCRPC patients of a prospective registry (NCT04833517), who were treated with [^177^Lu]Lu-PSMA-617 RLT and classified as early non-responders were included in this study. All patients received dual PET/CT imaging with [^18^F]FDG and [^68^Ga]Ga-PSMA-11 at baseline and after median two cycles of RLT. We tested potential biomarkers representing the “*change of glucometabolic activity (cGA)*” and “*change of glucometabolic activity in relation to PSMA expression (cGAP)*” composed of established parameters on [^18^F]FDG PET/CT as SUVmax, cumulative SUV of five lesions (SUV5), metabolic tumor volume (MTV) and total lesion glycolysis (TLG) and its corresponding parameters on [^68^Ga]Ga-PSMA-11 PET/CT, respectively, for association with overall survival (OS).

**Results:**

Kaplan–Meier analyses showed no significant association with OS for each tested cGA (cGA_SUVmax_*p* = 0.904, cGA_SUV5_, *p* = 0.747 cGA_MTV_*p* = 0.682 and cGA_TLG_*p* = 0.700), likewise the dual imaging biomarkers cGAP_SUVmax_ (*p* = 0.136), cGAP_SUV5_ (*p* = 0.097), and cGAP_TV_ (*p* = 0.113) failed significance. In contrast, cGAP_TL_, which is based on TLG and total lesion PSMA (TLP) showed a significant association with OS (*p* = 0.004). Low cGAP_TL_ (cut-off 0.7) was associated with significant longer survival (17.6 vs. 12.9 months).

**Conclusion:**

The novel biomarker cGAP_TL_, which represents the temporal change of whole-body TLG normalized by TLP, predicts overall survival in the challenging cohort of patients non-responding to PSMA-RLT.

## Background

Prostate cancer (PC) is among the most abundant solid malignant tumor diseases in men worldwide with a considerable mortality rate (1). Frequently, PC is progressing into a metastatic state that is resistant to physical or pharmaceutical castration by androgen deprivation therapy (ADT). This metastatic castration resistant prostate cancer (mCRPC) is associated to a poor prognosis (2–4). Commonly applied treatment options are, e.g., novel androgen axis drugs (NAAD) (5, 6), chemotherapy (7, 8), Ra-223 treatment (9), and PARP inhibitors (10). A further promising and previously approved treatment option is the prostate-specific membrane antigen (PSMA) directed radioligand therapy (RLT) using the beta-emitter ^177^Lu (in form of [^177^Lu]Lu-PSMA-617). While this therapy form has been shown to be effective and safe in several studies, a certain proportion of patients do not or insufficiently respond to PSMA-RLT (11–17). The assessment of response to therapy is commonly performed by evaluation of serum prostate-specific antigen (PSA) as a biochemical marker and by molecular imaging via PSMA-targeted positron emission tomography/computational tomography (PET/CT) e.g. [^68^Ga]Ga-PSMA-11 PET/CT. However, there is an unmet need for a further characterization of non-responding patients. The early prediction of outcome for the individual patient is essential, especially for patients with insufficient or no response to [^177^Lu]Lu-PSMA-617 RLT. The additional value of a [^18^F]fluorodeoxyglucose ([^18^F]FDG) PET/CT, that is performed, e.g., supplementary to [^68^Ga]Ga-PSMA-11 PET/CT in form of dual-tracer imaging is still controversial (18–21). The proposed value of [^18^F]FDG PET/CT in monitoring of mCRPC patients is suspected in its ability to characterize the state of dedifferentiation of tumor cells. With ongoing progression of the disease, tumor cells of mCRPC tend to change the expression profile of proteins on the cell surface, commonly including an upregulation of glucose transporter 1 (GLUT1) to meet the tumor cells higher demand for glucose, which results from an intensified energy metabolism by glycolysis (22). To date, it is an ongoing objective of clinical research to assess the role of [^18^F]FDG PET/CT and combined dual tracer PET/CT in characterizing the tumor profile and predicting the outcome for individual patients undergoing RLT.

With a focus on future clinical application, the aim of this study was to identify appropriate prognostic dynamic parameters derived from baseline and follow-up [^18^F]FDG PET/CT and dual-tracer imaging PET/CT for monitoring non-responding mCRPC patients undergoing PSMA-RLT.

## Methods

### Study design and patients

**Figure 1:**
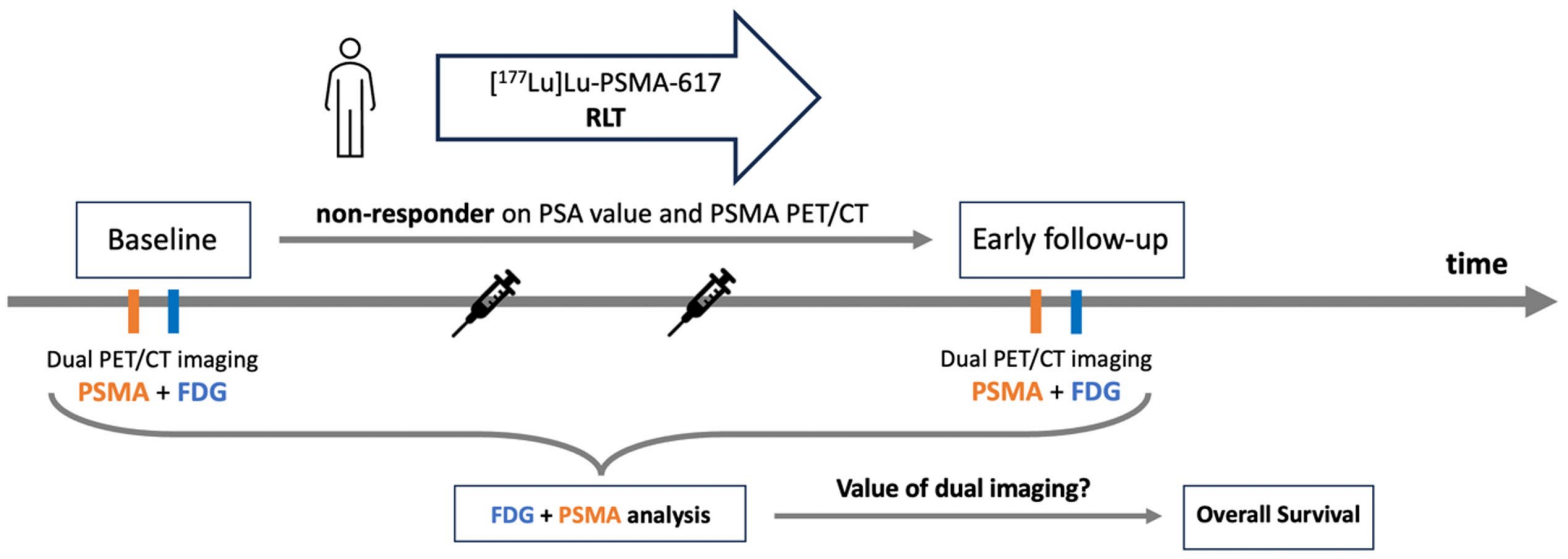
Study design.

This study involved *n* = 23 patients of the “*prospective registry to assess outcome and toxicity of targeted radionuclide therapy in patients with mCRPC in clinical routine*” *(REALITY Study),* NCT04833517, who were treated with [^177^Lu]Lu-PSMA-617 RLT classified as early non-responders. All patients received dual [^68^Ga]Ga-PSMA-11 PET/CT and [^18^F]FDG PET/CT imaging at baseline and at interim after one or two cycles of [^177^Lu]Lu-PSMA-617 RLT. Included patients experienced neither biochemical response nor molecular imaging response on [^68^Ga]Ga-PSMA-11 imaging according to commonly used criteria (23, 24). The mean PSA increase from baseline to interim was 56 ± 112%. To assess the value of [^18^F]FDG and dual imaging monitoring in these patients, PET metrics were obtained at baseline and follow-up, the respective data and derived dynamic parameters were analyzed for association with OS. The study design is depicted schematically in Figure 1.

All patients of the cohort received prior treatment including chemotherapy, NAAD or ADT. Summarized patient characteristics are presented in Table 1. Informed consent was obtained from all patients involved in this study and was conducted according to the guidelines of the declaration of Helsinki. PSMA-RLT was performed in consensus to the German Pharmaceutical Act §13 (2b). The analysis was approved by the local Institutional Review Board (ethics committee permission number 140/17).

**Table 1:** Patient characteristics.

Patient characteristics	Value
Age	
Median in [years], (range)	71 (59–85)
Age ≥ 65 years, *n* (%)	17 (73.9)
Age < 65 years, *n* (%)	6 (26.1)
PSA at baseline, in [ng/mL]	
Median (range)	109 (1–1956)
ALP, in [U/L]	
Median (range)	180 (53–748)
Hemoglobin, in [g/dL]	
Median (range)	11 (8–15)
< 13 g/dL, *n* (%)	17 (73.9)
ECOG performance status, *n* (%)	
0	4 (17.4)
1	12 (52.2)
≥2	7 (30.4)
Sites of metastases, n (%)	
Bone	20 (86.9)
Lymph node	17 (73.9)
Liver	7 (30.4)
Other	6 (26.1)
Prior therapies, *n* (%)	
Prostatectomy	11 (47.8)
Radiation	13 (56.5)
ADT	23 (100)
NAAD	22 (95.6)
Abiraterone	17 (73.9)
Enzalutamide	19 (82.6)
Abiraterone and Enzalutamide	14 (60.9)
Chemotherapy	19 (82.6)
Docetaxel	18 (78.3)
Cabazitaxel	11 (47.8)
Docetaxel and Cabazitaxel	10 (43.5)
[^223^Ra]Ra-dichloride	4 (17.4)

ADT, antiandrogen deprivation therapy; ALP, alkaline phosphatase; ECOG, Eastern Cooperative Oncology Group; NAAD, novel androgen axis drugs; PSA, prostate specific antigen.

### Treatment details

All patients included in the study received RLT with [^177^Lu]Lu-PSMA-617. Out of 23 patients, 6/23 patients received one cycle and 17/23 patient received two cycles of [^177^Lu]Lu-PSMA-617 until follow up imaging procedure. For the first cycle, mean activity of 7.6 ± 2.9 GBq was applied, for the second cycle the mean activity was 7.6 ± 1.3 GBq. For patients who received two cycles of [^177^Lu]Lu-PSMA-617 RLT, time-interval between both cycles was 6 ± 2 weeks. If two cycles were administered, the median cumulative activity was 13.4 ± 5.1 GBq. Administered [^177^Lu]Lu-PSMA-617 was synthesized following the recommended standard procedure (25). The ligand PSMA-617 was obtained from ABX advanced biochemical compounds GmbH (Radeberg, Germany), ^177^Lu was purchased from IDB Holland BV (Baarle-Nassau, Netherlands). Each patient received an intravenous infusion of 500 mL 0.9% NaCl, 30 min prior to treatment, as well as a cooling of salivary glands. Infusion of [^177^Lu]Lu-PSMA-617 was administered intravenously over a time-period of about 1 h.

### PET acquisition

Dual imaging by [^18^F]FDG PET/CT and [^68^Ga]Ga-PSMA-11 PET/CT was carried out in a short interval prior to start and after the first or second cycle of PSMA-RLT. Each patient received a baseline dual imaging procedure 3 ± 3 weeks before the first [^177^Lu]Lu-PSMA-617 RLT cycle was administered. The mean time between the two PET/CT scans at baseline was 6 ± 9 d. Dual imaging was repeated 8 ± 6 weeks after the first or second cycle. At follow-up the mean time between the two PET/CT scans was 6 ± 8 d. The total time between baseline and follow-up scans was 4 ± 3 months. For [^18^F]FDG and [^68^Ga]Ga-PSMA-11 PET/CT scans mean activity was 255.5 MBq ± 38.0 MBq and 138.5 MBq ± 20.7 MBq, respectively. Administration of tracer was followed by infusion of 500 mL 0.9% NaCl. [^18^F]FDG was deployed by ZAG (Karlsruhe, Germany). ^68^Ga was obtained from Eckert & Ziegler Strahlen-und Medizintechnik AG (Berlin, Germany) using a ^68^Ga/^68^Ge generator. The ligand PSMA-11 was provided via ABX advanced biochemical compounds GmbH (Radeberg, Germany). Following the recent imaging guidelines (26, 27), time-span between injection and imaging was 60 min for both PET scans. All PET/CT scans were conducted using a Biograph 40 mCT PET/CT scanner (Siemens Medical Solutions, Knoxville, TN, United States). Applied slice thickness was 3.00 mm, the PET acquisition was performed from vertex to mid-femur with 3 min/bed position for [^68^Ga]Ga-PSMA-11 and 2 min for [^18^F]FDG. The extended field of view was 21.4 cm (TrueV). PET reconstruction was achieved using a three-dimensional OSEM algorithm with 3 iterations, 24 subsets, Gaussian filtering, and a slice thickness of 5.0 mm. Decay correction, scatter correction, attenuation correction, and random correction were applied. For anatomic localization and attenuation correction, low-dose CT was attained with an X-ray tube voltage of 120 keV and modulation of the tube current using CARE Dose4D with a reference tube current of 50 mAs. The CT scans were reconstructed with a 512 × 512 matrix, applying an increment of 3.0 mm and a slice thickness of 5.0 mm.

### PET analyses and statistics

**Figure 2:**
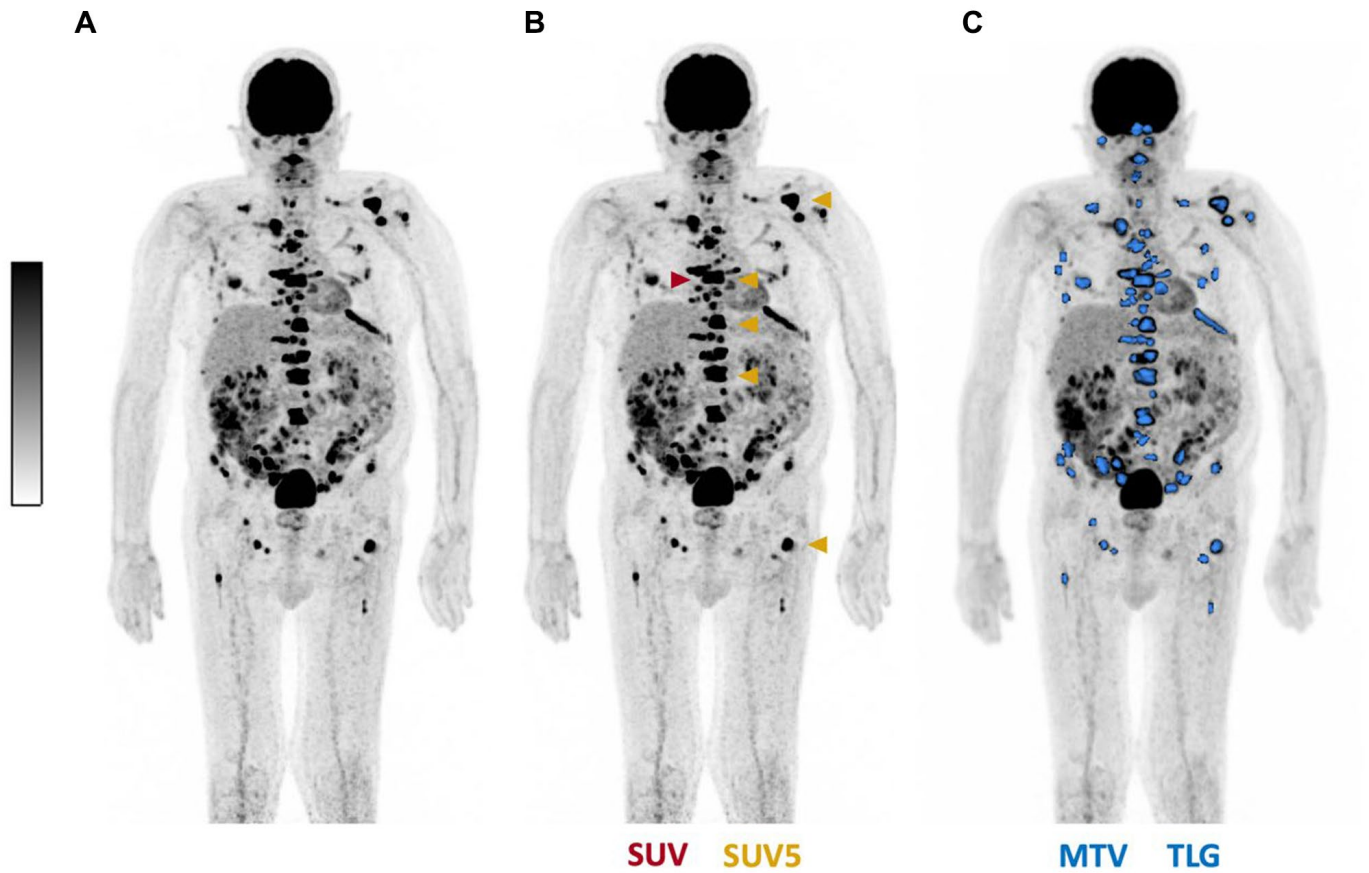
Representative example illustrating PET-derived parameters. **(A)** Maximum intensity projection of [^18^F]FDG PET/CT, displayed in **(B)** SUVmax (red), SUV5 (gold) and in **(C)** total tumor segmentation (blue) for calculation of MTV and TLG.

For [^18^F]FDG PET/CT four established parameters for use in were assessed at baseline and follow-up: (a) the maximum standard uptake value (SUVmax) (b) the cumulative SUV of the lesions with the most intensive uptake (SUV5) (c) the total metabolic tumor volume (MTV) and (d) the total lesion glycolysis (TLG) (28, 29). Quantitative analyses of each parameter was performed by Syngo.Via software (Siemens Medical Solutions, Knoxville, TN, United States). For calculation of MTV and TLG a semi-automatic tumor segmentation was used with a 41% threshold of SUVmax (27). MTV was calculated by the sum of the volume of each tumor lesion. TLG was determined as the summed products of volume and uptake (SUVmean) of all tumor lesions. Figure 2 exemplifies the derived parameters.

Based on the four described imaging parameters, different biomarker were introduced to assess the change over time. We introduced the “*change of glucometabolic activity*” (cGA), which is defined as the ratio between the follow-up and the baseline value of the respective imaging parameter. The cGA was calculated for SUV, SUV5, MTV, and TLG.

In addition, for each parameter we introduced and analyzed a corresponding dual imaging biomarker to assess the change in both [^18^F]FDG and [^68^Ga]Ga-PSMA-11 PET/CT over time. This dual imaging biomarker, “*change of glucometabolic activity per PSMA expression*” (cGAP) was defined as the relative change of the ratio between the [^18^F]FDG and its comparable [^68^Ga]Ga-PSMA-11 imaging parameter. The comparable parameters of MTV and TLG were total PSMA tumor volume (PSMA-TV) and total lesion PSMA (TLP), respectively. PSMA-TV and TLP were calculated according to Ferdinandus et al. (30).

Finally, two groups were segregated by the median of the respective value and tested for association with overall survival (OS) by Kaplan–Meier method and log rank test. OS was defined as interval starting at first image acquisition and terminated either by the occurrence of death or last contact. Cut-off date of the study was 05th July 2023. All statistics were calculated using the PRISM version 8.2.0 (GraphPad software, San Diego, United States) or SPSS version 29 (IBM Corp., Armonk, United States). A *p*-value < 0.05 was defined as statistically significant.

## Results

At baseline, SUVmax and SUV5 values, derived from [^18^F]FDG PET/CT were 12.7 ± 8.5 and 50.0 ± 36.9. Follow-up values were 11.9 ± 8.0 for SUV and 45.9 ± 33.0 for SUV5. The baseline values for the parameter of MTV and TLG were 314.0 ± 318.6 mL and 1588.8 ± 1967.5 mL x SUV. On follow-up imaging, values of 357.0 ± 381.6 mL and 1544.3 ± 1781.3 mL x SUV were found for MTV and TLG, respectively. Comprehensive information of baseline and follow-up imaging parameters is presented in Supplementary Table S1.

Deriving from baseline and follow up [^18^F]FDG PET/CT, calculation of cGA_SUVmax_ and cGA_SUV5_ resulted in median values of 0.884 (range 0.404–2.045) and 0.909 (range 0.222–1.565), respectively, while calculation of cGA_MTV_ and cGA_TLG_ yielded median values of 1.023 (range 0.129–5) and 1.098 (range 0.128–5,701), respectively.

**Figure 3:**
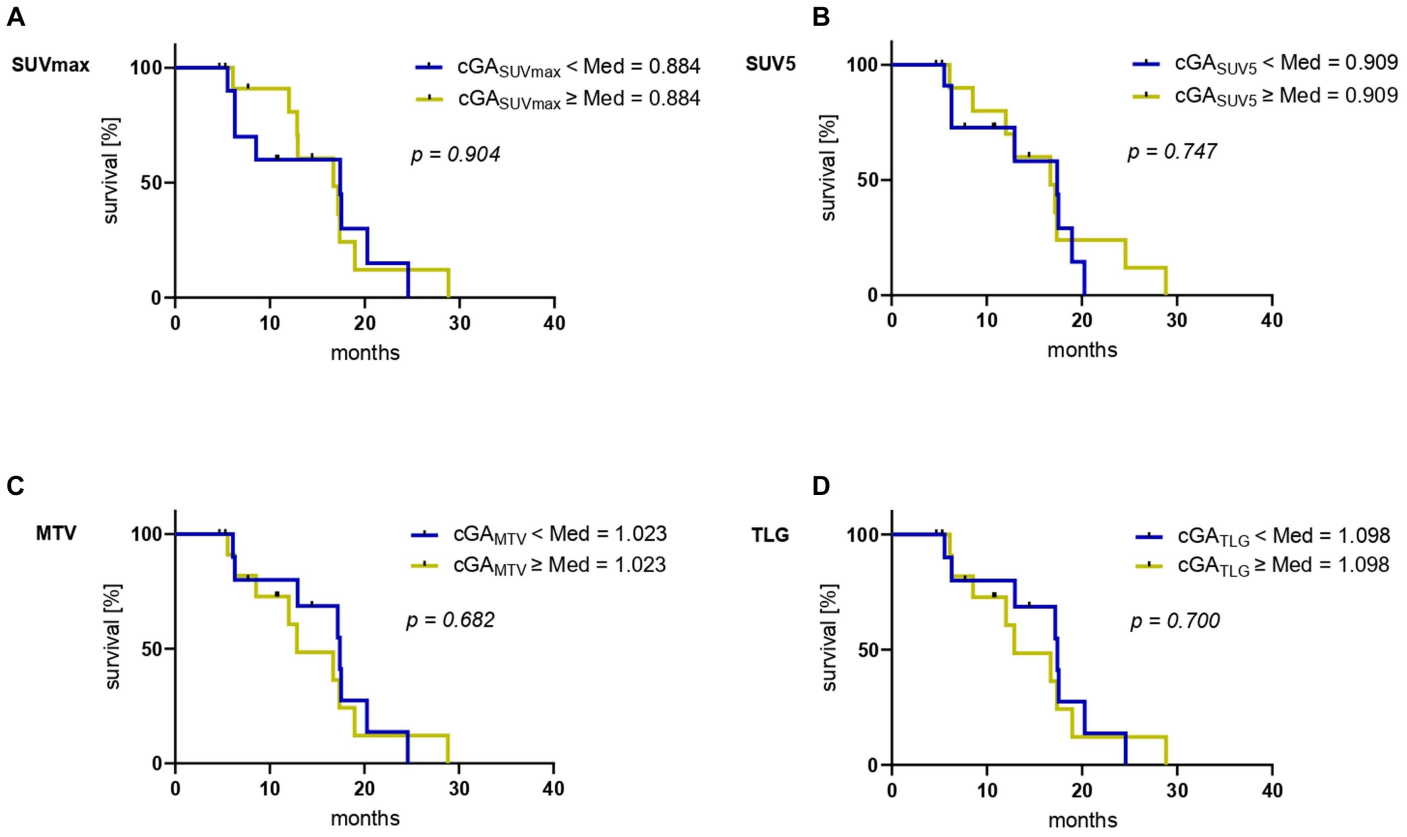
Kaplan–Meier curves for overall survival (OS) stratified by the median of the respective “change of glucometabolic activity” (cGA) **(A)** cGA_SUVmax_, **(B)** cGA_SUV5_, **(C)** cGA_MTV_ and **(D)** cGA_TLG_.

The median OS for the observed cohort was 17.2 months (CI 11.9–22.5 months). Kaplan–Meier analyses stratified by the median value of each cGA are depicted in Figure 3. No significant association with OS was observed for cGA_SUVmax_ (*p* = 0.904 Figure 3A), cGA_SUV5_ (*p* = 0.747 Figure 3B), cGA_MTV_ (*p* = 0.682 Figure 3C), nor cGA_TLG_ (*p* = 0.700 Figure 3D). Table 2 comprises detailed information on survival analyses. Similarly, the corresponding parameters derived from [^68^Ga]Ga-PSMA-11 PET/CT did not reach level of significance in this cohort (Supplementary Figure S1).

**Table 2:** Survival analysis.

Group	*n*	Median OS (m)	95% CI lower threshold	95% CI upper threshold	*p* value
Overall	23	17.2	11.9	22.5	–
cGA_SUVmax_	23	17.2	11.9	22.5	0.904
< Med (0.884)	11	17.4	0.00	37.6	–
≥ Med (0.884)	12	16.7	11.1	22.3	–
cGA_SUV5_	23	17.2	11.9	22.5	0.747
< Med (0.909)	11	17.4	6.7	28.3	**–**
≥ Med (0.909)	12	17.2	11.6	22.5	–
cGA_MTV_	23	17.2	11.9	22.5	0.682
< Med (1.023)	11	17.4	11.8	23.0	**–**
≥ Med (1.023)	12	12.9	6.6	19.2	**–**
cGA_TLG_	23	17.2	11.9	22.5	0.700
< Med (1.098)	11	17.4	11.8	23.0	**–**
≥ Med (1.098)	12	12.9	6.6	19.2	**–**
cGAP_SUVmax_	23	17.2	11.9	22.5	0.136
< Med (1.114)	11	17.6	4.6	30.5	**–**
≥ Med (1.114)	12	16.7	12.1	21.3	**–**
cGAP_SUV5_	23	17.2	11.9	22.5	0.097
< Med (0.970)	11	17.6	10.8	24.3	**–**
≥ Med (0.970)	12	16.7	11.1	22.3	–
cGAP_TV_	23	17.2	11.9	22.5	0.113
< Med (0.828)	11	17.4	17.2	17.7	**–**
≥ Med (0.828)	12	12.9	7.5	18.3	**–**
cGAP_TL_	23	17.2	11.9	22.5	**0.004**
< Med (0.700)	11	17.6	17.2	17.9	**–**
≥ Med (0.700)	12	12.9	7.4	18.4	–

cGA, change of glucometabolic activity; cGAP, change of glucometabolic activity per PSMA expression.

**Figure 4:**
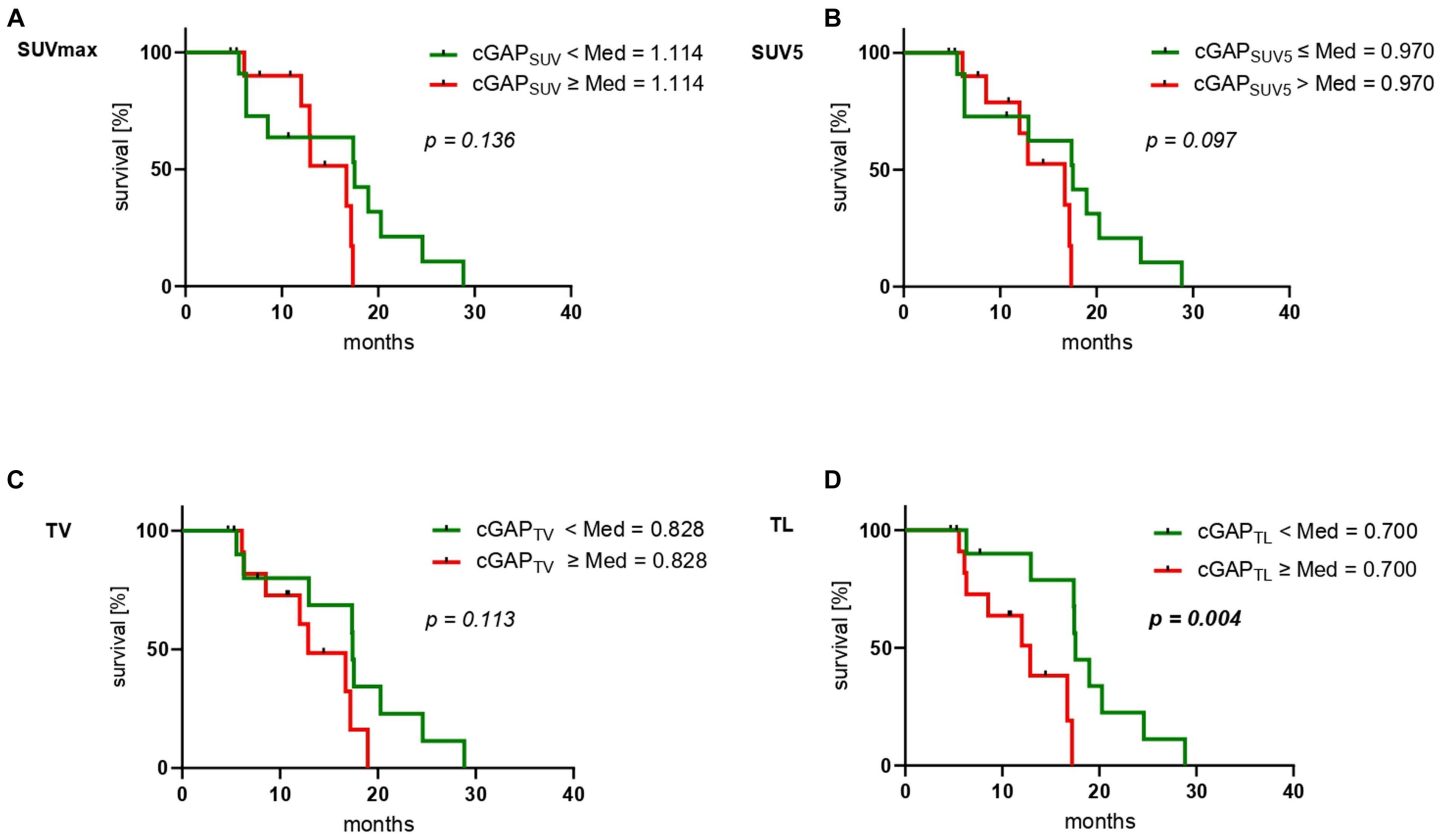
Kaplan–Meier curves for overall survival (OS) stratified by the median of the respective “change of glucometabolic activity per PSMA expression” (cGAP) **(A)** cGAP_SUVmax_, **(B)** cGAP_SUV5_, **(C)** cGAP_TV_ and **(D)** cGAP_TL_.

**Figure 5:**
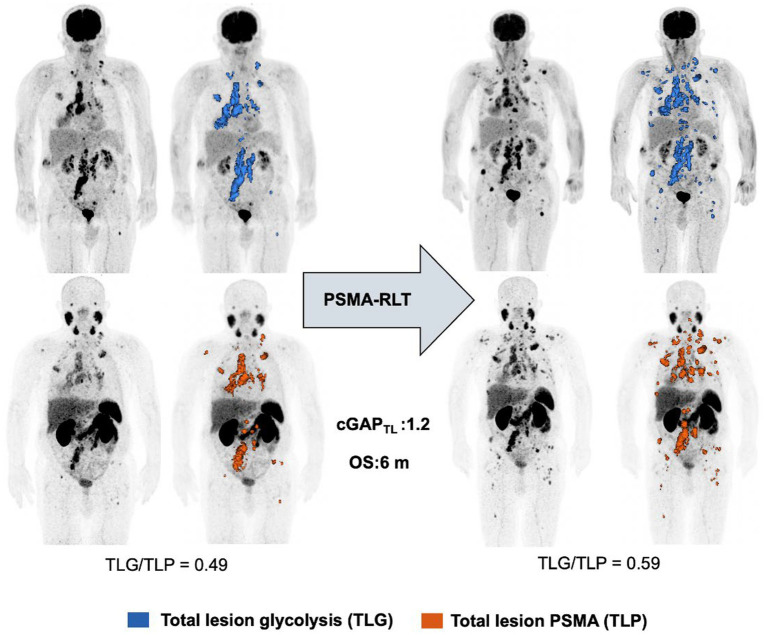
Exemplary patient demonstrating high cGAP_TL_ level.

**Figure 6:**
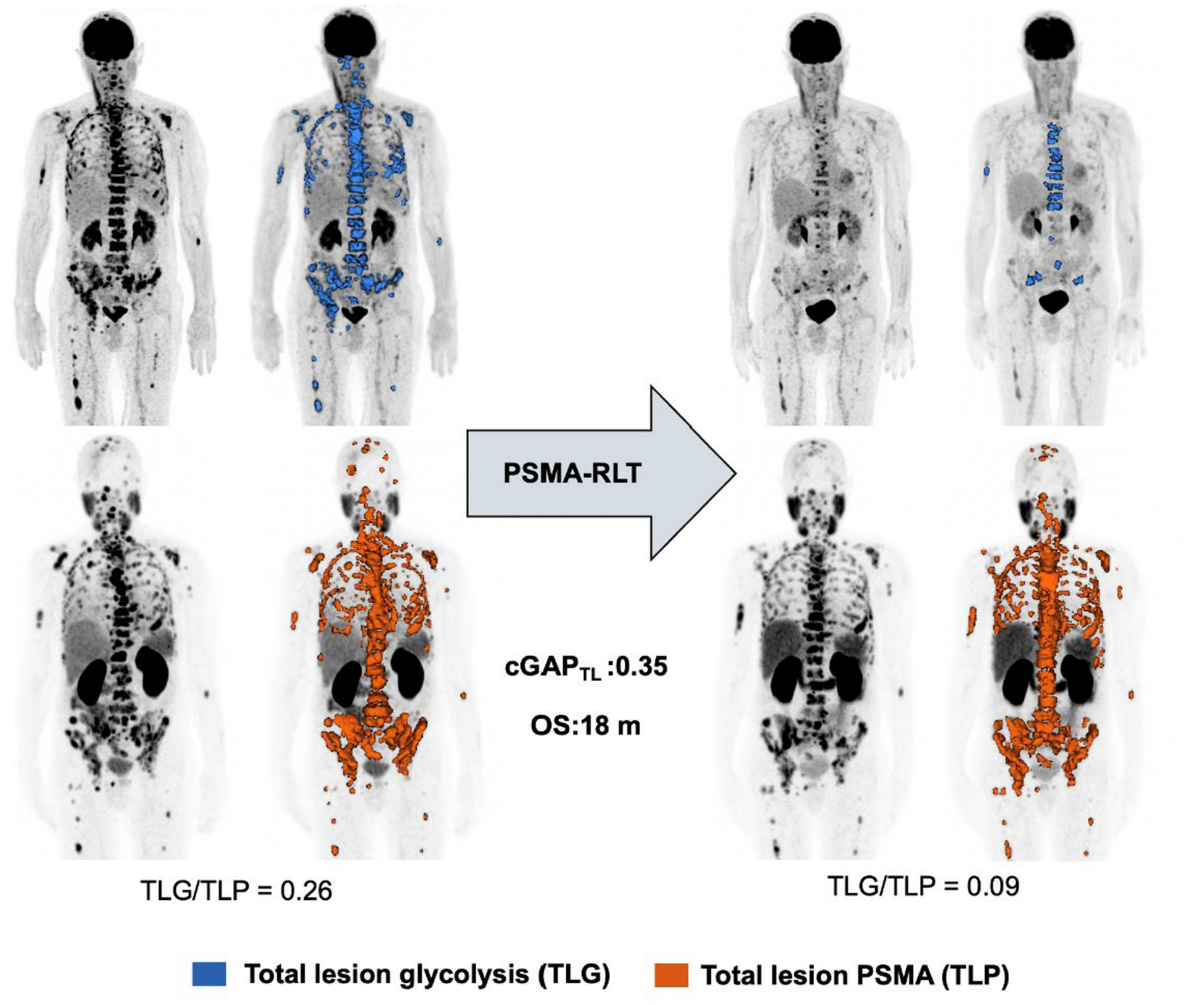
Exemplary patient demonstrating low cGAP_TL_ level.

Deriving from dual imaging baseline and follow up [^18^F]FDG and [^68^Ga]Ga-PSMA-11 PET/CT, calculation of cGAP_SUVmax_ and cGAP_SUV5_ yielded median values of 1.114 (range 0.394–6.46) and 0.970 (range 0.256–1.380), respectively. For cGAP_MTV_ and cGAP_TL_ median values of 0.828 (range 0.089–3.980) and 0.700 (range 0.087–2.760) were determined. Figure 4 shows Kaplan–Meier analyses stratified by the median value for the different cGAP. Neither cGAP_SUVmax_ (*p* = 0.136 Figure 4A), cGAP_SUV5_ (*p* = 0.097 Figure 4B), nor cGAP_TV_ (*p* = 0.113 Figure 4C) reached the level of significance. In contrast, statistically significant association with OS was observed for cGAP_TL_ (*p* = 0.004 Figure 4D). Patients with a low cGAP_TL_ (cut-off 0.7) experience a significant longer survival (median OS 17.6 months, CI: 17.2–17.9 months) than patients with a high cGAP_TL_ (median OS 12.9 months, CI: 7.4–18.4 months). Dual imaging [^18^F]FDG and [^68^Ga]Ga-PSMA-11 PET/CT of two exemplary patients with high and low cGAP_TL_, respectively, is shown in Figures 5, 6.

## Discussion

Despite the known high response rate of [^177^Lu]Lu-PSMA-617 RLT (1, 2), there is a considerable number of patients who do not or only insufficiently respond to this therapy (31, 32). Even in this group of non-responders, there are large inter-individual heterogeneities with different course of disease and survival resulting in a high demand for biomarkers predicting these individual courses. To our knowledge, this is the first study investigating biomarkers derived from periodic dual [^18^F]FDG and [^68^Ga]Ga-PSMA-11 PET/CT imaging during PSMA-RLT. Herein, we found that a new biomarker “*change of glucometabolic activity per PSMA expression for total lesions*” (cGAP_TL_), representing the dynamic change of whole-body lesion glycolysis (TLG) normalized to whole-body lesion PSMA (TLP), reliably predicts overall survival in this challenging cohort of patients not responding to [^177^Lu]Lu-PSMA-617 RLT.

The subgroup with low cGAP_TL_ (cut-off 0.7) demonstrated a significantly longer OS (*p* = 0.004) than the subgroup with a high cGAP_TL_. The cutoff used in this study was the median cGAP_TL_ in our cohort. This means that patients showing a decrease of total tumor glycolytic activity of more than 30% per total tumor PSMA (i.e., PSMA-based total tumor burden) experience significantly longer survival despite the non-responding character (after max. 2 cycles of RLT) of their disease. The introduced temporal dual imaging biomarker cGAP_TL_ appears to be superior to the other dual imaging parameters tested, such as cGAP_SUVmax_, cGAP_SUV5_ or cGAP_TV_ with regard to OS (each *p* > 0.09). The superiority of cGAP_TL_ is presumably based on the combination of both, uptake and tumor volume, whereas the other parameters only consider one of each.

Glucose metabolism in relation to PSMA expression may reflect a prognostically adverse aggressive metabolic feature of mCRPC lesions. Preclinical data suggests that dedifferentiated prostate carcinoma cells with intense GLUT1 expression are related to enhanced proliferation and aggressiveness of disease, which is commonly associated with shorter survival (33, 34). Hence, we speculated that a temporal increase of glycolytic activity normalized by PSMA expression, may indicate development towards a more aggressive nature of the disease accompanied by potential dedifferentiation, irrespective of disease extent and would thus represent a predictive biomarker. In particular, our study showed that patients who have a substantial decrease in total tumor glucometabolic activity normalized by PSMA expression have a relatively favorable prognosis despite failing early response. Surprisingly, in contrast to the above-mentioned dual imaging biomarker, none of the tested single imaging parameters depending exclusively on [^18^F]FDG PET/CT imaging (cGA_SUVmax_, cGA_SUV5_, cGA_MTV_ and cGA_TLG_) were significantly associated with OS (all *p* > 0.6) in our analysis. To our knowledge, no study has yet investigated [^18^F]FDG PET/CT imaging as a monitoring tool for PSMA-RLT.

However, there are several previous studies demonstrating the prognostic value of [^18^F]FDG PET/CT imaging at baseline prior initiation of PSMA-RLT in mCRPC (28, 30, 35–37). In particular, Ferdinandus and colleagues reported shorter survival of patients with high MTV at baseline (30), while Bauckneht et al. demonstrated that MTV, but also TLG at baseline predict OS (28). Recently, the secondary outcome analysis of an open-label, randomized phase II trial (TheraP) reported that MTV, derived from [^18^F]FDG PET/CT was prognostic for OS (38). These studies emphasize the potential role of [^18^F]FDG PET/CT in the management of mCRPC patients. The cGAP_TL_ presented in this study combines information about the phenotypic cancer profile regarding their GLUT1 and PSMA expression while additionally considering the treatment-associated change over time. This compound parameter of the relationship between both, glucose metabolism and PSMA expression, and time course may explain its highly predictive nature regarding OS. In line with these results, a study based on experimental and bioinformatic methods by Bauckneht et al. reported that [^18^F]FDG and [^68^Ga]Ga-PSMA-11 PET/CT seem to provide complementary and independent prognostic information (39). This study highlights the value of combined PET/CT scans in providing early information on the risk of progression. Similarly, the dual imaging parameter cGAP_TL_ might help characterize patients with insufficient early response to RLT with regard to potential treatment adjustment. Possible treatment options include augmentation with [^225^Ac]Ac-PSMA-617 or chemotherapy. Rational decision-making during RLT, especially in case of progression, represents a challenge for physicians and remains an important topic of research. This fits into the context of treatment optimization by personalized medicine taking into account tumor heterogeneity and potential promising treatment options for each individual (18, 40). Comprehensive monitoring via molecular imaging, including the use of predictive biomarkers, might certainly contribute to this approach. While implementation of the relatively complex parameter of cGAP_TL_ into clinical practice seems to be challenging, the foreseeable improvements and increasing integration of AI tools in software should enable its convenient use in the future. Dual imaging with [^18^F]FDG and [^68^Ga]Ga-PSMA-11 PET/CT and derived molecular imaging parameters merit further investigation in larger future studies, ideally in a prospective setting, to confirm and extend our findings.

The results of this study have to be seen in light of some limitations. Firstly, the study suffers from its retrospective nature and its small number of patients, which certainly can impact the results. Secondly, while the composition of the considered cohort was purposely pre-selected with patients who did not adequately respond to [^177^Lu]Lu-PSMA-617 RLT, a generalization of our results is limited. Studies are recommended in larger unselected cohorts before generalization of results is legitimate. Another point to consider is the potential bias, which might rise from the non-uniform timespan between baseline and interim scan, as well as the differing number of the administered cycles of the radiopharmaceutical. Due to the large number of metastatic lesions, it was not feasible to analyze them individually. In this context, AI may help to address this issue in future studies.

## Conclusion

The here introduced novel biomarker “*change of glucometabolic activity per PSMA expression*” (cGAP_TL_), which represents the temporal change of total lesion glycolysis (TLG) normalized by total lesion PSMA (TLP), predicts overall survival in the challenging patient cohort non-responding to [^177^Lu]Lu-PSMA-617 RLT. Monitoring by dual molecular imaging with [^18^F]FDG and [^68^Ga]Ga-PSMA-11 PET/CT may thus prove valuable in mCRPC patients undergoing PSMA-RLT.

## Data availability statement

The original contributions presented in the study are included in the article/Supplementary material, further inquiries can be directed to the corresponding author.

## Ethics statement

The studies involving humans were approved by Ärztekammer des Saarlandes, Homburg, Germany. The studies were conducted in accordance with the local legislation and institutional requirements. The participants provided their written informed consent to participate in this study. Written informed consent was obtained from the individual(s) for the publication of any potentially identifiable images or data included in this article.

## Author contributions

CB: Data curation, Investigation, Writing – original draft, Writing – review & editing. JE: Data curation, Formal analysis, Investigation, Visualization, Writing – original draft. AB: Formal analysis, Investigation, Software, Writing – original draft, Writing – review & editing. MB: Formal analysis, Methodology, Resources, Supervision, Writing – review & editing. SM: Conceptualization, Data curation, Investigation, Methodology, Writing – review & editing. AS-S: Methodology, Project administration, Supervision, Writing – review & editing. FK: Supervision, Validation, Visualization, Writing – review & editing. SE: Conceptualization, Investigation, Project administration, Supervision, Validation, Writing – original draft, Writing – review & editing. FR: Data curation, Formal analysis, Investigation, Methodology, Project administration, Supervision, Writing – original draft, Writing – review & editing.
