# Supplementary material for: Change of glucometabolic activity per PSMA expression predicts survival in mCRPC patients non-responding to PSMA radioligand therapy: introducing a novel dual imaging biomarker
Source: Front Med (Lausanne). 2024 Jan 17;10:1339160. doi: 10.3389/fmed.2023.1339160 (PMC10827880; doi:10.3389/fmed.2023.1339160)
Supplement: Supplementary file 1 [file Table_1.DOCX]

**Supplements**

**Table (S1)** *Data of baseline and follow-up imaging parameters.*

|  | Median | Min. | Max. | Mean | SD |
| --- | --- | --- | --- | --- | --- |
| **[^18^F]FDG PET/CT**  Baseline |  |  |  |  |  |
| SUVmax | 10 | 5 | 37 | 12.7 | 8.5 |
| SUV5 | 41 | 5 | 160 | 50.0 | 36.9 |
| MTV [mL] | 202 | 11 | 1082 | 314.0 | 318.6 |
| TLG  Follow up | 1020 | 43 | 7356 | 1588.8 | 1967.5 |
| SUVmax | 8 | 5 | 33 | 11.9 | 8.5 |
| SUV5 | 31 | 17 | 122 | 45.9 | 33.0 |
| MTV [mL] | 205 | 10 | 1260 | 357.0 | 381.6 |
| TLG | 637 | 40 | 5441 | 1544.3 | 1781.3 |
| **[^68^Ga]Ga-PSMA11 PET/CT**  Baseline |  |  |  |  |  |
| SUVmax | 36 | 16 | 141 | 45.7 | 33.8 |
| SUV5 | 131 | 48 | 285 | 139.3 | 71.4 |
| PSMA-TV [mL] | 1365 | 18 | 4717 | 1460.5 | 1413.8 |
| TLP  Follow up | 7561 | 70 | 47762 | 11475.3 | 12875.9 |
| SUVmax | 28 | 7 | 92 | 33.6 | 17.5 |
| SUV5 | 98 | 23 | 353 | 128.0 | 70.9 |
| PSMA-TV [mL] | 1257 | 19 | 4260 | 1650.7 | 1417.9 |
| TLP | 7863 | 75 | 52346 | 12323.5 | 12885.8 |
|  |  |  |  |  |  |
| **cGA** |  |  |  |  |  |
| cGA_SUVmax_ | 0.884 | 0.404 | 2.045 | 1.016 | 0.422 |
| cGA_SUV5_ | 0.909 | 0.222 | 1.565 | 0.937 | 0.367 |
| cGA_MTV_ | 1.023 | 0.129 | 5.000 | 1.383 | 1.212 |
| cGA_TLG_ | 1.098 | 0.128 | 5.701 | 1.279 | 1.188 |
| **cPE** |  |  |  |  |  |
| cPE_SUVmax_ | 0.869 | 0.271 | 1.96 | 0.934 | 0.428 |
| cPE_SUV5_ | 0.872 | 0.483 | 2.049 | 0.964 | 0.36 |
| cPE_MTV_ | 1.197 | 0.837 | 6.527 | 1.665 | 1.374 |
| cPE_TLP_ | 1.131 | 0.788 | 6.319 | 1.618 | 1.31 |
| **cGAP** |  |  |  |  |  |
| cGAP_SUVmax_ | 1.114 | 0.394 | 6.460 | 1.318 | 1.222 |
| cGPAP_SUV5_ | 0.970 | 0.256 | 1.380 | 1.104 | 0.590 |
| cGAP_TV_ | 0.828 | 0.089 | 3.980 | 1.038 | 0.805 |
| cGAP_TL_ | 0.700 | 0.087 | 2.76 | 0.896 | 0.730 |

cGA, change of glucometabolic activity; cGAP, change of glucometabolic activity per PSMA expression; cPE, change of PSMA expression

**Figure (S1)** *Kaplan-Meier curves for overall survival (OS) stratified by the median of the ‘change of PSMA expression’ (cPE) defined as the ratio between the follow-up and the baseline value of the respective imaging parameter:* ***A:*** *cPE_SUVmax_,* ***B:*** *cPE_SUV5_,* ***C:*** *cPE_MTV_ and* ***D:*** *cPE_TLP_.*
